# Internal dynamics within primary care teams in two Spanish regions during the COVID-19 pandemic: a qualitative study

**DOI:** 10.1186/s12875-022-01674-2

**Published:** 2022-03-31

**Authors:** Montserrat Pulido-Fuentes, Ana María Palmar-Santos, Juan Antonio Flores-Martos, Carmen Cipriano-Crespo, Laura Alicia Rubio, Luisa Abad González, MVictoria Navarta-Sánchez

**Affiliations:** 1 Faculty of Health Sciences, University of Castilla-La Mancha, Talavera de La Reina, Spain Avenida Real Fábrica de Sedas S/N, 45600 ToledoTalavera de la Reina, Toledo, Spain; 2 Departamento de Enfermería, Universidad Autónoma de Madrid, Calle Arzobispo Morcillo nº 4, 28029 Madrid, Spain; 3 Faculty of Social Science, University of Castilla -La Mancha, 45600 Talavera de la Reina, Toledo, Spain; 4 Faculty of Education Sciences and Humanities, University of Castilla -La Mancha, 16071 Cuenca, Spain

**Keywords:** COVID-19, Emerging infectious diseasees, Primary care teams, Healthcare workers, Pandemic, Qualitative study, Teamwork

## Abstract

**Background:**

Pandemics and epidemics have represented public health emergencies with severe consequences at a global level. Primary care teams have played a crucial role in disease surveillance and monitoring during the COVID-19 pandemic through early detection, contact tracing, and isolation of positive cases. The objective of this study was to explore the impact of the COVID-19 pandemic on primary care teams regarding their internal dynamics and their professional performance.

**Methods:**

Qualitative study carried out between July and December 2020 in two large central and southern Spanish regions (Castilla la Mancha and Madrid). Semi-structured interviews and focus groups were conducted with primary care workers. Data was analysed using thematic content analysis. Participants were accessed using purposive sampling.

**Results:**

A total of 53 primary care workers participated in the study, of which 38 were individually interviewed, and 15 participated in three focus groups.The analysis of their experiences revealed two main themes regarding the impact of the COVID-19 pandemic on primary care teams: *1) The need to reorganise traditional roles:* Primary care settings closed their doors to the public and their workers restructured their roles to ensure the delivery of essential services; 2) *The need to implement a new primary care delivery model*: Each primary care team had to self-organise, making sure their reference population was cared for and developing resource optimisation strategies.

**Conclusions:**

Primary care teams have quickly adapted their roles and internal dynamics to respond to the demands generated by COVID-19. In the new delivery model, some positive aspects could be highlighted – such as increased communication between professionals and the use of telemedicine for some cases. However, it is important to address the negative impact that the COVID-19 crisis has had on of the main functions of primary care. These measures are necessary to promote well-being in primary care teams, and to provide quality care that addresses the complex and individual needs of each person and reduces inequalities in healthcare delivery.

## Introduction

The COVID-19 pandemic that began in early 2020 has caused an international crisis causing more tan 5.7 million deaths and 395 million infections worldwide, with Europe being the continent with the highest number of infections. Spain, in particular, has been one of the hardest-hit European countries, ranking tenth worldwide and fifth in Europe in number of deaths [1, 2]. As a result of exceptional levels of demand, the Spanish healthcare system was put under extreme strain – a situation sustained throughout the whole of the pandemic.

Primary care settings are the "front door" of healthcare systems and have played a crucial role in the surveillance and monitoring of COVID-19. Together with public health teams, they have contributed to the study and identification of at-risk populations and the early detection of complications, intervening in different stages of the health–disease process [3–5] and monitoring the chronically ill. The WHO has pointed out from the outset the importance of maintaining the capacity of primary care settings to continue delivering essential services during the pandemic, as well as monitoring self-isolating cases [6]. In this regard, it is recommended that primary care professionals should be involved in planning and action for health emergency risk management [7].

In the Spanish context, Primary care settings were totally or partially closed down at the start of the pandemic – with reductions in the numbers of consultations and work shifts. With a pandemic not yet under control and the impending threat of new surges, or indeed new pandemics, it has been necessary to implement changes in the structure of primary care delivery. These have impacted the internal dynamics within PCTs and their professional performance.

Primary care teams (PCTs) are organisational structures based on multi-professional units that include healthcare workers – i.e., general practitioners (GPs), paediatricians, nurses, midwives, pharmacists – and non-clinical staff –i.e., social or administrative workers – and are based on specific primary care centres. These teams work mainly with a focus on public health and health promotion, based on area health diagnoses [8]. As with many other processes during the COVID-19 pandemic, these units had to adapt in response to this health crisis, which caused changes in their organisation [9–11]. Spanish PCTs have been responsible for detection and screening tasks and monitoring COVID-19 patients self-isolating at home [12]. This has required drastic transformations in the allocation of the human and material resources available in primary care settings at short notice – due to rapid surges in cases during COVID-19 outbreaks [13].

During the pandemic, many countries have promoted new strategies within PCTs, such as the adoption of new technologies and telemedicine as an alternative to in-person consultations [3] – with telephone visits acquiring a central role to minimise face-to-face contact for primary care delivery. One consequence of this, however, was a drastic increase in administrative tasks [6, 14]. Simultaneously, the concentration of already-limited resources on potential and critical COVID-19 cases caused delays and interruptions in routine outpatient consultations and planned interventions, with medical personnel sometimes being redeployed to cover different clinical services [15, 16]. Problems accessing the health system – particularly primary care – and delivering high-quality patient care during the COVID-19 pandemic have been recognised worldwide [17–19]. As a result, many countries have had to implement significant transformations to their healthcare systems to respond to the COVID-19 crisis [11].

A number of studies have addressed changes experienced in primary care and, in particular, PCTs during the COVID-19 pandemic from the viewpoint of their increased workload or measuring different quality indicators of patient care service [20, 21]. However, only a few studies have focused on the viewpoint of the different team members in PCTs. This is crucial to assessing the response to the COVID-19 health crisis and to identifying strategies to help navigate this pandemic [22] while maintaining a high-quality, resilient healthcare system [23] – optimising primary care resources to cope with the current, and future, pandemics. This study explored the impact of the COVID-19 pandemic on PCTs’ internal dynamics and professional performance.

## Methods

### Design

This is a qualitative exploratory study [24, 25] based on the analysis of participants' experiences. This approach allows to inductively explore personal experiences of the primary care teams during the COVID-19 pandemic.

### Participants

The study participants were primary care workers from two Public Healthcare Services in Spain: the Castilla-La Mancha Healthcare Service (SESCAM) and the Madrid Region Healthcare Service (SERMAS).These regions were selected for the study due to the similarity of their profiles in terms of incidence during the first surge of the COVID-19 pandemic, management of healthcare resources, restrictions in access to the healthcare system, and economic and socio-demographic factors [26, 27].The sample included clinical and non-clinical professionals working in public primary care settings – community-based centres offering non-specialised care: family medicine, paediatrics, nursing, physiotherapy, midwifery and perinatal health, dentistry, emergency health technicians, administrative staff, social workers, orderlies, cleaners, and collection of laboratory samples. Purposive sampling was used to include different participants and increase diversity in the results [28]. In order to guarantee maximum variability in the experiences collected, the following criteria were considered in the sampling: demographic profiles (gender and age), employment status (permanent, temporary, zero-hours), professional roles (practice managers, general practitioners, paediatricians), and years of experience (under or over ten years of experience). Another factor taken into account was whether these workers had dependent family members (Table 1). Participants were invited to the study through a previous online survey study as part of an observational study. Invitations to participate in the study were sent via institutional e-mails to more than 600 healthcare professionals working in the regional healthcare systems where the study was conducted (purposive sampling). Participants had to answer questions regarding their health status, job characteristics, sociodemographic profiles, and the Burnout Clinical Subtype Questionnaire (BCSQ-36). Once the survey was completed, participants were asked whether they wanted to join the qualitative research phase, either via individual interviews or focus group discussions. A total of 677 invitations were sent, of which only 37% resulted in acceptance to join the project. From this 37% (*n* = 252), only 22% (*n* = 56) agreed to participate in the qualitative phase. We also used snowball sampling, with initial participants identifying additional subjects among their contacts [29]. Those who expressed their willingness to participate in an individual interview or focus group and fitted the target profile were sent an e-mail with information on the study’s aims, anonymisation, and personal data processing procedures. The research team responded to queries from the participants via e-mail or telephone calls.

**Table 1:** Profiles of the study participants

	Number of Participants
Male/Female	16/37
Rural/Urban	21/32
Over/Under 10 Years of Experience	38/15
With/Without Dependent Family Members	27/26
Nurses	26
General Practitioners	2
Nursing Manager	5
Practice Manager	4
Nursing Aides	3
Emergency Technicians	2
Social Workers	3
Physiotherapists	3
Administrative Staff	2
Midwives	2
Cleaners	1

### Data collection

Data were collected through in-depth, semi-structured interviews [30, 31] and focus groups [32, 33], designed to gain a broad perspective on the views of primary care teams, which were conducted between July and December 2020.July 2020 was a period characterised by an initial easing of COVID-19 restrictions, and with it the arrival of the "new normal", which offered an apparent improvement. There included a progressive easing of the strict mobility limitation measures and Spain entered a scenario of "pandemic control". Although there was a generalised rise in the number of infections in Spainour country, along with the rest of Europe, in a second pandemic wave (November 2020) that affectedmore young people, quantitatively there were fewer people hospitalised and less deaths in all age groups than in the first wave. This was accompanied by a surge in postponed demands from primary care patients [34–36]. Primary care centres were working under tremendous strain due to increased workload, while face-to-face consultations were still mostly unavailable.

First, a total of 38 individual interviews were conducted with primary care workers, with durations of between 45–70 min each, using an interview guide (Table 2). Using semi-structured interviews, the study inductively explored the personal experiences of primary care workers during the COVID-19 pandemic – an approach that allowed us to register and integrate into the analysis the voices of these professionals as individuals with agency, examining their experiences during this period. Most individual interviews were completed in-person, in venues selected by the participants themselves, although some took place via telephone or video calls.

**Table 2:** Interview Guide

Subject Areas	Questions
Working in a primary care setting	• Describe a working day before and during the COVID-19 pandemic • How has the health crisis affected your internal dynamics, working environment and professional performance, compared to the situation before the pandemic? • How have the work management practices and care provision planning been affected? • What communication channels have been used regarding information, training, protocols?
Working as part of a team	• Describe how the relationship with the rest of the team has been. Has anything changed during this time?
Healthcare provision	• How has the health crisis affected users, patients, families, the community, and primary care in general, compared to the situation before the pandemic?
	• Would you like to add something else?

Second, to obtain an overview of the subject studied from the dialogue between participants, three focus groups were carried out with a total of 15 primary care workers. With the focus groups, we were able to examine the more collective and shared social discourse on the impacts and effects of COVID management on work dynamics among professionals working for the same regional health service, but in different primary care health centres. Focus groups were led by one moderator (a research team member) helped by another researcher (an observer). They followed a previously defined guide (Table 2) and had durations of between 60–120 min each.

Interviews and focus groups were conducted by different members of the research team and were audio-recorded. All the researchers had extensive experience in qualitative designs, research, and qualitative analysis. A fieldwork diary was used to record contextual issues and the researchers’ observations and thoughts [37]. Ten participants withdrew on the day of the interview or focus group due to pandemic-related issues. None of the researchers involved in data collection was work-related to the study subjects.

### Data analysis

Both individual interviews and focus groups were transcribed verbatim. An inductive thematic content analysis of the participants' experiences was conducted, identifying emerging codes, categories, and themes [38].

Participants were assigned an alphanumeric code used for data logging, category creation, and as a reference for literal quotes extracted from the accounts.

Each researcher analysed the data independently, establishing initial codes and categories. The results of their individual analyses were discussed in several team meetings, where final codes, categories, and themes were agreed upon through consensus between all researchers. Contrasting different perspectives on the same study subject guarantees the quality of the results.

All participants were offered the opportunity to review the audio or written records and the subsequent analysis, via an email sent by the member of the research team who conducted the interview, to confirm the interpretation of their narratives established by the researchers. In addition, the COREQ guidelines for reporting qualitative research [39] were followed to ensure the study's quality.

### Ethical considerations

The study was approved by the corresponding ethics committees in each region (Castilla La Mancha: ref. 23/2020; Madrid:ref.34–20). The research followed the ethical principles outlined in the 1964 Declaration of Helsinki and the Belmont Report. Data collected from interviews and focus groups were handled in line with current guidelines on the ethical implications of research, and anonymised in line with current data protection laws [40]. All subjects provided informed consent to their participation in the study.

All data were treated with due confidentiality and in line with the General Data Protection Regulation (EU) 2016/679 of the European Parliament and of the Council of 27 April 2016, on the protection of natural persons with regard to the processing of personal data and on the free movement of such data (GDPR), and the Spanish Organic Law 3/2018, of 5 December, on personal data protection and guarantee of digital rights (LOPDGDD). Only members of the research team had access to data collected through interviews and focus groups.

## Results

A total of 53 Primary care workers, the majority of them healthcare professionals (78.4%), female (72.5%), and with more than ten years of work experience in this area (70.5%) participated in this study. Of these, 38 were individually interviewed and 15 participated in three focus groups.

The analysis of their experiences revealed two main themes associated with the impact of the COVID-19 pandemic on the internal dynamics within PCTs: 1) The need to reorganise traditional roles, and 2) The need to implement a new primary care delivery model (Table 3).

**Table 3:** Shows literal excerpts from the interviews and focus groups

**Theme 1. The need to reorganise traditional roles**	
**Category 1.1** Emergence of teamwork	*Nobody stopped at just their responsibilities – you always did more and tried harder, it was a moral imperative – we all disinfected ceilings, walls… We've done all sorts – we have worked together, the rigid structure of tiers was gone – I have felt very supported, everyone involved themselves fully (RUF-25, physiotherapist)* *At first, we were all rowing in the same direction, a fantastic team, cohesive, really united – however, over time it started to crack, and you could feel it, because it was getting heavy – I mean, everything COVID is heavy, and it affects all the teams, the good ones and those where relationships were not so good or were poor (Nurse, focus group-3)*
**Category 1.2** Triage: a problem for PCTs	*There was a strange feeling regarding the door, because at the beginning it was shut for good – like "nobody is coming in here", without really knowing what we were to do – because we've closed the door, and now what? (Nurse, focus group- 3)* *We were told "you have to organise a triage", so we brought a table downstairs, we organised some shifts, and so on. And everybody had to make do as well as they could – the lack of protection, of resources, because you almost had to find your own resources – (UN-24, nurse)* *You are more alert now, I spend my days asking things I should not know about – I am not a healthcare professional, but I need to ask people about COVID symptoms so I can refer them to my colleagues (UAD-6, administrative staff)* *In the hospital, everything is more guarded… There is an access control point, with a security guard, which is important, and janitors to take your temperature and limit access for accompanying persons. The hospital is more organised, a lot more, here we had to do it… Primary care hasn't* – *well, it hasn't been as well looked after as it should have been (UN-13, nurse)*
**Category 1.3** “Everything is COVID”: Some responsibilities neglected	*It has been let down, yes… Health education was something we did a lot of, programs about smoking, about polymedication* – *we used to do small group meetings, perhaps once a year for each subject, or sessions with information stands near the door, but all that had to stop, of course* – *we haven't done any of that (UN-4, nurse)* *I can do nothing, or rather, I do what I do, but I feel that so much passes me by… I cannot work properly like this* – *it feels like we just muddle through (RM-21, practice manager)* *My main takeaway is that nursing in primary care is a giant with feet of clay – our activity went under, we stopped doing all the things we used to do, despite these being what we really should have done… Also the feeling of unity, because in my team there was unity, the first months we were all in it together – but then, we should be looking out for patients who are at home, having a hard time, losing relatives, or with hospitalised relatives that they cannot phone – we have to do it. We haven't been able to do it, although it is something we should have done (Nurse focus group-3)*
**Theme 2**. **The need to implement a new primary care delivery model**	
**Category 2.1** Self-management in primary care centres	*The feeling was… that we mostly had to rely on ourselves (UN-14, nurse)* *Our only resources were those of the health centre – nothing more (UM-23, practice manager)* *It is very sad to have people waiting in the street […] it rained, and people were there with their umbrellas, in the street, a lady with her crutch, a man with a walking frame. Really, it is very upsetting. But there wasn’t enough time to start thinking "let's see what we can do to sort this out, let's see what we can do, because in two days it's going to be icy" (Nurse, focus group-3)*
**Category 2.2** The limits of telephone consultations	*There will be two kinds of patients, the smart ones and the rest – and either they survive, they adapt, or they are going to be left behind (Nurse, focus group-1)* *The way they come through the door, how they move, their agility, the abilities they possess – I am already doing a neurological assessment. [With telephone consultations] you lose people's spontaneity when they ask for something that they might not even know what it is, and when you see them, when you examine them, sometimes you find issues that were not what they were asking about to start with. So you are missing that freshness, the possibilities it opens for diagnosis… You have to make the most of technology for what it is worth, but it should not be an excuse, nor a limit or a shield to protect us. Medicine is about direct engagement with the patients, and there is no other way around it (RM-20, practice manager)*
**Category 2.3** Pandemic opportunities	*It has helped to identify things that we were doing wrong… It is not necessary to check the blood pressure every month, or give appointments for repeat prescriptions… We have identified things that we were not doing correctly (UN-8, nursing manager)* *To be honest, I am working a lot better now… Of course, now I have half an hour for each patient, which before I did not have. […] The pandemic has been beneficial for me – I can do my work a lot better, because I have the time – Now I can say that what I do is truly useful, that I know what I am doing (UF-6, physiotherapist)* *[…] and we have noticed that primary care is always prescribing specialist referrals, many of them unnecessary – but the specialists do not cancel them, and the population have high expectations and care demands – the ophthalmologist says I have to be checked every six months but it has been more than I year, I am very worried – but you can see that they are alright (UM-20, practice manager)*

### Theme 1. The need to reorganise traditional roles

The need to reorganise traditional roles Primary care delivery underwent substantial transformations during the COVID-19 pandemic, affecting the internal dynamics within PCTs. At the pandemic's start, primary care settings closed their doors to the public to limit infectious exposure. In-person and home visits were put on hold as primary care practices turned to telephone consultations, which required a restructuring of roles to ensure the delivery of essential services (Table 3). Several categories were identified regarding this subject:

#### Emergence of teamwork

During the pandemic, workers in PCTs saw their usual routines – conducting their tasks individually while being part of a wider team – interrupted. Instead, team colleagues had to reorganise all their day-to-day tasks to address the new challenges imposed by the pandemic, while assuming new responsibilities that were sometimes not entirely suited to their professional roles. On the one hand, this fostered a strengthening of relationships and mutual support among workers. With the initial chaos and uncertainty, new and more frequent communications and increased interactions were introduced – i.e., daily meetings, breakfast gatherings, or mobile chat groups to keep up to date. The mutual support within the teams also allowed them to cope better with work-related stress.

As the months went by, however, cracks started to appear *–* triggered by tiredness and the lack of an end being in sight *–* and individualist attitudes re-emerged. This gradually exhausted the workers' endurance and organisational abilities, with discomfort settling in their work environment and permeating the teams.

#### Triage: a problem for PCTs

Changes in professional roles and boundaries also caused physical transformations within the primary care settings. As opposed to the pre-pandemic “open centre” approach, a triage system was introduced to control access to those centres that remained open during the pandemic – since many of these centres, especially local clinics, were closed. However, access criteria differed for each of the primary care settings analysed in this study – criteria were not uniform, and they were implemented by a varying number of non-medical staff, with different professional categories. This is a key aspect, since patients were referred to different primary care services based on the result of their triage assessment.

The triage at the main door of the primary care centres involved different workers, affecting their internal dynamics and contributing to an increase in occupational stress.

At the same time, as our participants noted, the management and control of the front door required bringing additional staff into the teams to ensure their safety – using specialist care delivery as a reference.

#### “Everything is COVID”: some responsibilities neglected

As a result of the implementation of triage and other pandemic-related tasks, some primary care responsibilities became neglected. Among the activities that had to be put on hold, our participants mentioned health promotion programs, preventive healthcare, health education, and chronic illness care.

The cracks starting to appear in healthcare provision can be appreciated in an increase in guilt – healthcare workers blaming themselves, thinking they are not doing their job correctly.

Our research revealed the impact of the pandemic on PCTs, with some responsibilities and agendas being overridden. The reorganisation of responsibilities due to the health emergency made some workers keener to assert their professional role.

### Theme 2. The need to implement a new primary care delivery model

In order to tackle the COVID-19 crisis, primary care settings were forced to reorganise their care delivery model at short notice (Table 3). Regarding this issue, several subcategories were identified:

#### Self-management in primary care centres

There was a general perception among our study participants that primary healthcare – both staff and medical practices – had been abandoned. Each setting had to reorganise and self-manage as a small island, and workers found it difficult to share their experiences and problems due to lack of communication.

Improvisation and trial-and-error were the main strategies to navigate day-to-day challenges. Our participants suggested that primary care acted as a dam containing the pandemic, to keep the hospitals from collapsing. There was general confusion about where people could go to access healthcare, when, and how. Some received care in their homes, via phone calls, others outside the medical practices, or even in their cars. The centres themselves remained empty and were perceived as dangerous places.

#### The limits of telephone consultations

According to the primary care workers participating in our study, their priorities were making sure their reference population was cared for, keeping some kind of “contact”, and conducting basic monitoring tasks to meet other needs of their population aside from the health emergency. Professionals suggested that the new, post-COVID primary care model must reach even further, since the care delivery model implemented during the pandemic risked leaving behind precisely those population groups that usually find it harder to access the healthcare system.

In this sense, telephone consulting was considered beneficial for certain users and types of consultations. It was particularly useful for bureaucratic issues *–* i.e., the prescription of medicines, diagnostic tests, and fit for work/sick notes, or to refer potential COVID cases to a dedicated service.

However, some patients “got lost” with this approach *–* they did not answer the phone, never phoned again, gave up.

Telephone visits act as a filter, even as a barrier, as opposed to face-to-face consultations *–* which allow clinicians to identify needs that people are not always able to express, as one practice manager noted.

#### Pandemic opportunities

Their accounts underscored the opportunities that this “experience” offered to identify practices that could be improved *–* i.e., poor practices that had been normalised, without revision or re-assessment.

It has also highlighted types of consultations in which time allowance is critical. During the pandemic, by avoiding overcrowding the medical practices, consultations could take longer. Healthcare professionals were also more aware of the work that they were doing, avoiding duplications.

It also exposed the volume of specialist consultations that could be conducted in primary care, thus optimising resources and delivering higher-quality care.

## Discussion

The COVID-19 pandemic has had a significant impact in the internal roles and work dynamics within PCTs. Primary care workers quickly reorganised their roles and implemented a new primary care delivery model to respond to the unprecedented demands generated by the COVID-19.

### Reorganisation of traditional roles

The results of our study revealed that during the pandemic interactions between healthcare professionals increased, allowing them to keep up-to-date. There was also increased mutual support within teams, which allowed them to cope better with work-related stress. This results are in line with those published in a comparative study conducted in eight European countries, which underlined an increase in the moral support and informattion feedback among primary care teams to adapt to new work routines implemented during the pandemic. [41]. It would be positive to reinforce these strategies, to improve interdisciplinary teamwork and share care and responsibility for patients – which has been highlighted as a key element for primary care settings to deliver cost-effective and comprehensive care [42]. As noted by other authors, effective interprofessional teams [43] and optimising the increasing diversity of the primary care workforce [44] might make healthcare less labour-intensive [45]. Therefore, these are strategies that would allow tackling the challenges imposed on primary care by the COVID-19 pandemicand provide long-term benefits.

The results identified in this study of the Spanish context suggest that primary care professionals had to reorganise their roles within each centre and introduce new strategies – such as triage – without receiving adequate formation on its implementation. This situation has also been noted in other European countries [41]. Moreover, changes in the organisational structure and the work environment might be significant stress factors among co-workers [46, 47]. Primary care leaders should improve communication channels with their teams and provide regular practical information to reassess priorities and direction explicitly [43]. Similarly, coordination between health centres and with other sectors to create multisectoral actions is crucial to address the complex challenges of the population, improve their health and well-being, and promote sustainable development in healthcare systems [48].

### The impact of Covid-19 on primary care responsibilities and their workers

Our study showed that some primary care responsibilities became neglected due to the implementation of pandemic-related tasks. Among the activities that had to be put on hold were health promotion programs, preventive healthcare, health education, and care for chronic illnesses. Our results are in line with those published for other countries, which have revealed a dramatic reduction in the number of primary care consultations– which affected in particular the care for chronically ill patients [14, 49–52]. These findings suggest that the pandemic has impacted primary care service provision and the health of their reference population, particularly those with chronic conditions. Strategies to mitigate these impacts and improve primary care service provision should be a priority, specially regarding the consequences of pandemic restrictions on how the care for chronically ill patients was conducted [14, 49–52].

Our findings revealed that primary care workers experienced mental health challenges and feelings of guilt because they thought they were not doing their job correctly, since they have to give up their usual roles to devote themselves to COVID-related tasks. Recent research in other countries [46, 53] has highlighted that healthcare workers worldwide might experiment mental health problems because they are working under extreme pressures and making impossible decisions regarding resource allocation due to the COVID-19 pandemic. Also, it is essential to bear in mind that healthcare workers are at higher risk of contagious exposure [54], making them a vulnerable group [55]. Thus, primary care authorities should implement strategies to manage mental health challenges, guilt, and the burnout caused by dealing with a public health emergency [43, 46].

### Telemedicine

Our study participants noted that, during the pandemic, telephone consultations became the prevalent care delivery strategy. This was a crucial resource that helped them reduce exposure to contagion in primary care centres and manage their workload while maintaining contact with their reference population. This is in line with results of studies conducted in other countries, stressing the importance of adopting new technologies to mitigate the impact of COVID-19 on primary care services [14, 51, 56, 57]. Telemedicine has facilitated the delivery of safe remote triage processes and care continuity in some primary care services [56]. However, some concerns about the use of telemedicine with older adults, deprived population groups, and people with lower digital literacy have been noted, both in our study and others [49, 58, 59]. In addition, digital consultations do not allow for a number of procedures including routine physical examinations, and thus might mask potentially serious illnesses – with people not asking for an appointment until their situation is extremely serious [51, 50]. Thus, face-to-face consultations remain essential to carry out physical examinations that allow the early identification of serious diseases and to avoid inequalities in health care access in those groups who find virtual care difficult to manage [49–51, 58, 59]. Before the COVID-19 pandemic, the widespread adoption of remote consultations was fragmentary and insufficient, partly due to healthcare systems resisting the digital transition [10, 60]. Nevertheless, a healthcare workforce ready for telemedicine will have a protective effect both on professionals and users in future pandemics. Thus, primary care authorities should reinforce telemedicine with adequate training and investing in digital resources [51, 56, 61, 62].

### Limitations

The representation of different primary care workers in the study sample was unequal – 49% of the participants were female nurses, which might have biased the results obtained. Healthcare activity in primary care settings did not stop during the pandemic, and the care of patients with other diagnoses continued despite staffing issues due to COVID-19 infections. This situation could perhaps explain the low participation of some groups, in particular primary care physicians. It is also entirely possible that those professionals most willing to be included in the study were also more aware of problems in the primary care delivery model. However, this could also be a positive factor, since nurses play a crucial role in the management of primary care settings. Thus, we believe their opinions are relevant, and might suggest new avenues for primary care provision.

On the other hand, as far as we know this is the first study to explore the impact of the COVID-19 pandemic on primary care teams’ professional performance in Spain that includes the opinion of different healthcare workers. Future studies focusing on these issues are more relevant than ever before, if we are to understand how primary care has responded and adapted to the unprecedented health crisis COVID-19 has caused worldwide.

## Conclusions

Primary care teams have quickly adapted their roles to respond to users and the demands generated by COVID-19. The new care delivery model implemented in primary care has identified dynamics that are important to maintain and indeed reinforce, such as improving communications within teams and the use of telemedicine in certain situations. At the same time, it is important to address the impact that COVID-19 has had on the main roles of primary care– in particular, the neglect of care towards patients with chronic illnesses and the well-being of its workers. These strategies are necessary to guarantee a system that delivers high-quality care for its users, is safe for its workforce, and reduces inequalities in healthcare delivery.

## Data Availability

The datasets generated and/or analysed during the current study are not publicly available due to ethical issuess but are available from the corresponding author on reasonable request.

## Abbreviation

PCTs: Primary care teams
